# Comparison of low calorie high protein and low calorie standard protein diet on waist circumference of adults with visceral obesity and weight cycling

**DOI:** 10.1186/s13104-018-3781-z

**Published:** 2018-09-21

**Authors:** Fiastuti Witjaksono, Joan Jutamulia, Nagita Gianty Annisa, Septian Ika Prasetya, Fariz Nurwidya

**Affiliations:** Department of Nutrition, Faculty of Medicine Universitas Indonesia, Dr. Cipto Mangunkusumo Hospital, Jl. Salemba Raya No. 6, Jakarta, 10430 Indonesia

**Keywords:** Visceral obesity, Weight cycling, Waist circumference, Low-calorie diet, High-protein diet

## Abstract

**Objectives:**

Many individuals with visceral obesity who previously had succeeded in reducing body weight regain and this loss–gain cycle repeats several times which is called as weight cycling. We aimed to evaluate the effect of a low calorie high protein diet (HP) compared to a low calorie standard protein diet (SP) on waist circumference of visceral obese adults with history of weight cycling.

**Results:**

In this open-randomized clinical trial, participants were asked to follow dietary plan with reduction in daily caloric intake ranging from 500 to 1000 kcal from usual daily amount with minimum daily amount of 1000 kcal for 8 weeks and were divided in two groups: HP group with protein as 22–30% total calorie intake; and SP group with protein as 12–20% total calorie intake. There was a statistically significant difference (*P* < 0.001) between waist circumference before and after the dietary intervention among both groups. Meanwhile, there was no statistically significant difference in the mean reduction of waist circumference between HP and SP groups (*P* = 0.073). Taken together, the protein proportion does not significantly affected waist circumference.

*Trial registration* ClinicalTrials.gov NCT03374150, 11 December 2017

**Electronic supplementary material:**

The online version of this article (10.1186/s13104-018-3781-z) contains supplementary material, which is available to authorized users.

## Introduction

Obesity has become one of world’s biggest health problem. There were 1.9 billion (39%) overweight adults and 600 million (13%) adults with obesity in 2014 [1]. In Indonesia, the prevalence of overweight adult was 13.5% of the general population and the prevalence of obese adults were 15.4% of the general population [2].

Repeated cycles of body weight loss followed by subsequent weight gain are termed as weight cycling. Obese individuals with weight cycling history have higher risk of developing chronic disease due to increased fat mass in every cycle and a tendency to become central obesity [3, 4]. The excess of fat in visceral adipose tissue is known as visceral or abdominal obesity [5]. Individuals with excess visceral fat have a greater risk of developing insulin resistance, metabolic syndrome, arterial hypertension and cardiovascular diseases than individuals with excess subcutaneous fat [6, 7]. Visceral obesity is also associated with a longer hospital stay, increased infectious and non-infectious complications, and increased mortality in hospital [8]. Individuals with visceral obesity also have increased predisposition to cancers of colon [9], breast [10], and prostate [11].

A study in Europe showed that dietary programs with low glycemic index and low energy density may prevent visceral adiposity [12]. Another study in the United States showed the role of protein intake in weight loss program in which higher protein was associated with lower BMI, lower waist circumferences, and higher high-density lipoprotein (HDL) cholesterol [13].

Anthropometric measurement such as BMI is commonly used to determine obesity [14]. However, BMI alone has a low sensitivity to measure adiposity. Waist circumference, alone or in combination with BMI, has shown to be an accurate predictor of visceral fat [15]. To determine cut-offs of waist circumference, it is important to consider gender and races. Men store 20–30% of their body fat in visceral fat irrespective of obesity status [16], while women do not accumulate significant visceral fat until they reach moderate level of obesity [17]. As for races, evidence suggests that the cut-offs of waist circumference for Asian is 90 cm for men and 80 cm for women [18]. However, the implication of protein proportion in the waist circumference remains to be elucidated. The objective of this study is to evaluate the effect of a low calorie high protein diet compared to a low calorie standard protein diet on waist circumference in adults with visceral obesity.

## Main text

### Methods

This open-randomized clinical trial evaluates the effect of a low calorie high protein diet compared to a low calorie standard protein diet on anthropometric measurement. The subjects were obese Jakarta Governmental Employee who suffers weight cycling and has a complete recorded health data in the Department of Nutrition Faculty of Medicine Universitas Indonesia. These subjects were recruited by a consecutive sampling and were randomized to be enrolled in one of the two intervention groups. There are two intervention groups: the high protein (HP) group, and; standard protein (SP) group. To determine which group the participants are in, random number generation method was used to determine which number represented HP or SP group. Each participant took a numbered envelope filled with a group name which would determine the group they belong to. The study has been registered in clinicaltrials.gov with registration ID NCT03374150 and has been approved by the Ethical Committee of the Faculty of Medicine Universitas Indonesia (No. 237/UN2.F1/ETIK/2017).

The inclusion criteria for the participants were men or women aged more than 20 years old with BMI ranging from 25 to 35 kg/m^2^, with a history of weight cycling and signed the informed consent to participate. The exclusion criteria were diabetes mellitus, a history of gastrointestinal tract resection, hormonal disorders such as abnormal thyroid function, hormonal contraception user, menopause, and abnormal kidney function. In this study, weight cycling is defined as a history of weight loss ≥ 2 kg and regaining weight or exceeding its initial body weight at least twice in the last 5 years. The history of weight cycling firstly was obtained from self-reports, which later would be confirmed by nutritionists.

Two weeks before dietary intervention, participants were interviewed for 24-h food recall to determine baseline calorie intake based on food photo books issued by Individual Food Consumption Survey Team (Tim Survey Konsumsi Makanan Individu), the Ministry of Health, Indonesia. In addition, anthropometric measurement and 1.5 ml vein blood after 8 h of fasting were also taken. Waist circumference measurements were performed by two trained enumerators under direct supervision by the investigator. Based on the calorie intake from 24-h food recall, participants underwent reduction in the amount of daily calorie intake ranging from 500 to 1000 kcal from their usual daily intake with the lowest possible amount of daily caloric intake was set at 1000 kcal. Nutritional consultation regarding diet plan and were given about appropriate type and amount of food and the suitable cooking methods. Participants were advised to follow the dietary plan without any change in their daily physical activities.

Participants in HP group were given macronutrient source with a composition of 22–30% protein, 50–55% carbohydrate and 20–25% fat, while participants in SP group were given macronutrient source with a composition of 12–20% protein, 55–60% carbohydrates and 20–30% fats. The diet program was conducted in 8 weeks, and each participant is given a log-book to be filled with the food they have eaten in a day. Follow-ups were done by daily phone calls and weekly counselling to ensure participants’ adherence. After the dietary program has been completed, anthropometric measurements, including waist circumference, were conducted to evaluate the difference prior and after intervention.

Statistical analysis was performed using software SPSS version 20 (IBM Corporation, Chicago, IL, USA). After analyzing the distribution of the data, the mean difference of waist circumference before and after treatment was analyzed with paired t-test. Meanwhile, the mean difference of waist circumference reduction between HP and SP group was analyzed with independent t-test. *P* value < 0.05 is considered to be significant.

### Results

Initially, there were 61 subjects who were randomized to the two arms and received interventions, 30 in SP group and 31 participants in HP group, yet only 54 of them completed the 8-weeks of diet program. Furthermore, 4 subjects from HP group and 2 from SP group did not attend the measurement session after they had completed the program. Therefore, there were only 23 and 25 subjects from HP and SP group, respectively, in which the waist circumference data were available and were analysed. Characteristics of participants prior to dietary intervention are provided in Table 1, while the dietary profile of participants during the course of treatment is shown in Additional file 1: Table S1. During the treatment, some participants experienced lethargy and nausea, but there were no significant adverse events.

**Table 1:** The characteristics of subjects at prior to dietary intervention

Variable	High protein (n: 23)	Standard protein (n: 25)	*P*-value*
Age (year)	36.09 ± 9.13	31 (20–47)	0.04^m^
Gender (%)			
Male	4 (17%)	3 (12%)	0.69^f^
Female	19 (83%)	22 (88%)	
Body mass index (kg/m^2^)	29.75 ± 3.51	29.74 ± 2.62	0.99^t^
Waist circumference (cm)	96.00 ± 9.54	96.24 ± 6.92	0.93^t^
Number of weight cycling history (%)			
2–3 times	20 (87%)	18 (72%)	0.29^f^
4–5 times	3 (13%)	7 (28%)	

* Significant value was set at *P* < 0.05

^m^Mann–Whitney U test

^f^Fisher’s Exact Test

^t^Independent samples t-test

Table 2 shows mean difference of waist circumference prior and after the treatment. Paired t-test resulted in *P*-value < 0.001, which shows there is a statistically significant difference in the mean of waist circumference before and after the treatment. When separated by gender, reduction of waist circumference after the treatment is also seen.

**Table 2:** Waist circumference before and after completing the dietary intervention

Variable	Mean of waist circumference (cm)		*P*-value*
	Before intervention	After intervention	
Female participants (N:41)	95.19 ±8.21	89.42 ±8.82	< 0.001^LR^
Male participants (N:7)	100 (97–114)	94.03 ±6.49	< 0.001^LR^
Total participants (N:48)	96.13 ± 8.19	90.09 ± 8.62	< 0.001^LR^

* Significant value was set at P < 0.05

^LR^Linear regression

^m^Mann–Whitney U test

We further analyzed if there is a significant difference in waist circumference reduction between participants in HP and SP group. Independent t-test resulted in *P* value = 0.073 which shows that there is no statistically significant difference in waist circumference reduction between the two groups, although there was a tendency that there is more reduction in waist circumference with low-calorie standard protein diet (Table 3).

**Table 3:** Reduction of waist circumference in high-protein and standard-protein low calorie-diet groups

Variable	Waist circumference (cm)		*P*-value*
	HP group (N: 23)	SP group (N: 25)	
Before intervention	96.01 ± 9.54	96.24 ± 6.92	0.925^LR^
After intervention	90.79 ± 9.78	89.45 ± 7.55	0.596^LR^
Pre- and post-intervention mean difference significance	P < 0.001^p^	P < 0.001^p^	
Waist circumference reduction (cm)	5.22 ± 3.29	6.78 ± 2.50	0.069^LR^

* Significant value was set at P < 0.05

^LR^Linear regression

^p^Paired samples t-test

### Discussion

Eight weeks of low-calorie diet intervention resulted in waist circumference reduction. This finding shows that the dietary intervention could reduce visceral fat and may be used in the treatment or prevention of visceral obesity. However, protein composition in the diet plan does not have significant effect in waist circumference reduction. This finding further proposes that calorie restriction is the determining factor in waist circumference reduction rather than the protein composition in the diet. Meanwhile, a slightly lower reduction of waist circumference in HP group compared to SP group, might be caused by the slightly higher mean daily calorie intake in HP group.

Dietary plans with low energy density (ED) have been found to reduce visceral adiposity [3]. Coherent with our study, past studies have also found that reduced-calorie diet results in significant weight loss [19] and visceral fat loss [20], regardless of the macronutrients-carbohydrates, fats, or protein-composition. Another study done in type 2 diabetes mellitus patients also shows that energy-restricted diet resulted in significant weight loss, but there is no significant enhancement of weight loss in energy-restricted diet with either low or high protein composition [21].

However, this finding differs in daily dietary plan with no caloric restriction. Consumption of high protein diets in daily dietary plan may have several benefits. In non-calorie-restricted diets, higher-protein intake is associated with lower BMI and waist circumference [4]. High protein diets with protein intake 1.2–1.6 g/kg body weight/day with 26–30 g protein/meal also provide improvements in appetite and cardiovascular and metabolic features [22].

The presence of weight cycling history in both groups probably can be the cause of indifferent outcome among them. HP diet is said to reduce fat mass since it stimulate satiety. Weight cycling bearers tend to lack of control in hunger-satiety and tend to be binge eaters [23]. This may be correlated with the activity of adipocytes responsible for leptin generation which influence hunger-satiety, although further studies are needed in this field [24].

Conclusively, differences in protein proportion does not significantly affected waist circumference in adults with visceral obesity, regardless of the protein composition in the dietary plan. Future study is needed to reveal the best proportion between carbohydrate, fat and protein in order to obtain sufficient reduction in waist circumference. Therefore, calorie restricted diet could be suggested in the treatment of visceral obesity. Protein and other macronutrients composition could be adjusted on patient’s individual needs or habit.

## Limitations

The limitation of this study is the low compliance of its participants. Potential selection bias may occur because a significant amount of participants missing during the primary outcomes measurement. Moreover, there is also potential recall bias because some participants did not fill their log-book everyday. The participants of this study were predominantly female with moderate-to-high socioeconomic status, which may have better diet performance than the general population. Other limitation is the nature of open-randomized clinical trial in which researcher and subjects knew the intervention.

## Additional file

**Additional file 1: Table S1.** The dietary profile of the subjects during the course of the treatment. Comparison of mean daily caloric intake, mean protein proportion of total daily caloric intake, mean carbohydrate proportion of total daily caloric intake, mean fat proportion of total daily caloric intake and number of days with diet programme compliance in the high protein (HP) group and in the standard protein (SP) group.

## Abbreviations

BMI: body mass index
HDL: high-density lipoprotein
HP: high protein diet
SP: standard protein diet

## References

[1] World Health Organisation. Obesity: situation and trends. http://www.who.int/gho/ncd/risk_factors/obesity_text/en. Accessed 15 Sept 2017.

[2] Basic Health Research 2013. Council of Research and Development, Indonesian Ministry of Health. [*Badan Penelitian dan Pengembangan Kesehatan. Riset Kesehatan Dasar. Jakarta; Kementerian Kesehatan RI:2013*].

[3] Oetoro S, Makmun LH, Lukito W, Wijaya A (2014). Effect of a weight loss program on body composition and metabolic syndrome markers in obese weight cyclers. Indones J Intern Med..

[4] Rodin J, Radke-Sharpe N, Rebuffé-Scrive M, Greenwood MR (1990). Weight cycling and fat distribution. Int J Obes..

[5] Shuster A, Patlas M, Pinthus JH, Mourtzakis M (1009). The clinical importance of visceral adiposity: a critical review of methods for visceral adipose tissue analysis. Br J Radiol.

[6] Donohoe CL, Doyle SL, Reynolds JV (2011). Visceral adiposity, insulin resistance and cancer risk. Diabetol Metab Syndr..

[7] Fox CS, Massaro JM, Hoffmann U, Pou KM, Maurovich-Horvat P, Liu CY (2007). Abdominal visceral and subcutaneous adipose tissue compartments: association with metabolic risk factors in the framingham heart study. Circulation.

[8] Tsujinaka S, Konishi F, Kawamura YJ, Saito M, Tajima N, Tanaka O, Lefor AT (2008). Visceral obesity predicts surgical outcomes after laparoscopic colectomy for sigmoid colon cancer. Dis Colon Rectum.

[9] Oh TH, Byeon JS, Myung SJ, Yang SK, Choi KS, Chung JW, Kim B, Lee D, Byun JH, Jang SJ, Kim JH (2008). Visceral obesity as a risk factor for colorectal neoplasm. J Gastroenterol Hepatol.

[10] Schapira DV, Clark RA, Wolff PA, Jarrett AR, Kumar NB, Aziz NM (1994). Visceral obesity and breast cancer risk. Cancer.

[11] Hafe VP, Pina F, Pérez A, Tavares M, Barros H (2004). Visceral fat accumulation as a risk factor for prostate cancer. Obes Res.

[12] Romaguera D, Ängquist L, Du H, Jakobsen MU, Forouhi NG, Halkjær J (2011). Food composition of the diet in relation to changes in waist circumference adjusted for body mass index. PLoS ONE.

[13] Pasiakos SM, Lieberman HR, Fulgoni VL (2015). Higher-protein diets are associated with higher HDL cholesterol and lower BMI and waist circumference in US adults. J Nutr.

[14] Okorodudu DO, Jumean MF, Montori VM, Romero-Corral A, Somers VK, Erwin PJ (2010). Diagnostic performance of body mass index to identify obesity as defined by body adiposity: a systematic review and meta-analysis. Int J Obes..

[15] Grundy SM, Neeland IJ, Turer AT, Vega GL (2013). Waist circumference as measure of abdominal fat compartments. J Obes.

[16] Björntorp P (2000). Metabolic difference between visceral fat and subcutaneous abdominal fat. Diabetes Metab.

[17] Despres JP, Lemieux I, Bergeron J, Pibarot P, Mathieu P, Larose E (2008). Abdominal obesity and the metabolic syndrome: contribution to global cardiometabolic risk. Arterioscler Thromb Vasc Biol.

[18] Misra A, Vikram NK, Gupta R, Pandey RM, Wasir JS, Gupta VP (2006). Waist circumference cutoff points and action levels for Asian Indians for identification of abdominal obesity. Int. J. Obes..

[19] Sacks FM, Bray GA, Carey VJ, Smith SR, Ryan DH, Anton SD (2009). Comparison of weight-loss diets with different compositions of fat, protein, and carbohydrates. N Engl J Med.

[20] de Souza RJ, Bray GA, Carey VJ, Hall KD, LeBoff MS, Loria CM (2012). Effects of 4 weight-loss diets differing in fat, protein, and carbohydrate on fat mass, lean mass, visceral adipose tissue, and hepatic fat: results from the POUNDS LOST trial. Am J Clin Nutr.

[21] Luscombe ND, Clifton PM, Noakes M, Parker B, Wittert G (2002). Effects of energy-restricted diets containing increased protein on weight loss, resting energy expenditure, and the thermic effect of feeding in type 2 diabetes. Diabetes Care.

[22] Leidy HJ, Clifton PM, Astrup A, Wycherley TP, Westerterp-Plantenga MS, Luscombe-Marsh ND (2015). The role of protein in weight loss and maintenance. Am J Clin Nutr.

[23] Elfhag K, Rössner S (2005). Who succeeds in maintaining weight loss? A conceptual review of factors associated with weight loss maintenance and weight regain. Int Assoc Study Obes Obes Rev..

[24] Strohacker K, Carpenter KC, Brian K (2009). Consequences of weight cycling: an increase in disease risk?. Int J Exerc Sci..

