# Supplementary material for: Comparison of low calorie high protein and low calorie standard protein diet on waist circumference of adults with visceral obesity and weight cycling
Source: BMC Res Notes. 2018 Sep 21;11:674. doi: 10.1186/s13104-018-3781-z (PMC6150981; doi:10.1186/s13104-018-3781-z)
Supplement: Supplementary file 1 — Additional file 1: Table S1. The dietary profile of the subjects during the course of the treatment. Comparison of mean daily caloric intake, mean protein proportion of total daily caloric intake, mean carbohydrate proportion of total daily caloric intake, mean fat proportion of total daily caloric intake and number of days with diet programme compliance in the high protein (HP) group and in the standard protein (SP) group. [file 13104_2018_3781_MOESM1_ESM.docx]

**Table S1.** The dietary profile of the subjects during the course of the treatment

| **Variable** | **HP**  **(n: 23)** | **SP**  **(n: 25)** | ***P*-value^*^** | |
| --- | --- | --- | --- | --- |
| Mean daily caloric intake (kcal) | 1025.40 + 163.59 | 991.18 + 91.82 | 0.371^t^ |  |
| Mean protein proportion of total daily caloric intake (%) | 26.50 + 3.99 | 19.98 (16.54 – 27.48) | <0.001^m^ |  |
| Mean carbohydrate proportion of total daily caloric intake (%) | 42.27 + 5.74 | 51.93 + 4.33 | <0.001^t^ |  |
| Mean fat proportion of total daily caloric intake (%) | 24.40 + 3.59 | 20.91 + 3.49 | 0.001^t^ |  |
| Number of days with diet programme compliance | 21 + 12 | 24 + 1 | 0.363^t^ |  |

^*^significant value was set at P<0.05

^t^independent samples t-test

^m^Mann-Whitney test
